# A Randomized Controlled Trial on the Effects of 12 Weeks of Aerobic, Resistance, and Combined Exercises Training on the Serum Levels of Nesfatin-1, Irisin-1 and HOMA-IR

**DOI:** 10.3389/fphys.2020.562895

**Published:** 2020-10-16

**Authors:** Sasan Amanat, Ehsan Sinaei, Mohammad Panji, Reza MohammadporHodki, Zahra Bagheri-Hosseinabadi, Hadis Asadimehr, Mohammad Fararouei, Aria Dianatinasab

**Affiliations:** ^1^Department of Nutrition, School of Health, Larestan University of Medical Sciences, Larestan, Iran; ^2^Rehabilitation Sciences Research Center, Shiraz University of Medical Sciences, Shiraz, Iran; ^3^Department of Cell and Molecular Biology, Faculty of Biological Science, Kharazmi University, Tehran, Iran; ^4^Kashmar Center of Higher Health Education, Mashhad University of Medical Sciences, Mashhad, Iran; ^5^Department of Clinical Biochemistry, Faculty of Medicine, Rafsanjan University of Medical Sciences, Rafsanjan, Iran; ^6^Department of Clinical Nutrition, School of Nutrition & Food Sciences, Shiraz University of Medical Sciences, Shiraz, Iran; ^7^Department of Epidemiology, School of Public Health, Shiraz University of Medical Sciences, Shiraz, Iran; ^8^Department of Biochemistry, Student Research Committee, Shiraz University of Medical Sciences, Shiraz, Iran

**Keywords:** metabolic syndrome, irisin, HOMA-IR, exercise program, insulin, aerobic, resistance, nesfatin-1

## Abstract

**Background/objective:** This study aimed to investigate the impacts of a 12-week training of the aerobic exercise (AE), resistance exercise (RE), and combined exercise (CE) on the serum levels of nesfatin-1, irisin-1 and some other metabolic and anthropometric indices in overweight women with metabolic syndrome.

**Methods:** Sixty overweight women with metabolic syndrome were assigned equally into four groups: aerobic exercise (AE, *n* = 15), resistance exercise (RE, *n* = 15), combined exercise (CE, *n* = 15), and control (*n* = 15). All groups underwent 12 weeks of intervention. The study variables were measured before and 24 h after the intervention period.

**Results:** Twelve weeks of training resulted in an increase of irisin-1 in the AE and CE groups and nesfatin-1 in all the intervention groups. As expected, all the trained groups exhibited a positive alteration in anthropometric indices and lipid profile in comparison with the control group. Besides, compared with the control group, insulin resistance (based on the homeostatic model assessment) in AE (*p* = 0.022), RE (*p* = 0.032), and CE (*p* < 0.001) groups were reduced significantly. According to the observed changes in the measured indices, serum irisin-1 was significantly correlated with body weight, BMI, body fat percentage, fasting insulin, and HOMA-IR. However, with regard to nesfatin-1, only a negative correlation was observed with body fat percentage and LDL-cholesterol.

**Conclusions:** The 12-week systematic training program changed circulating irisin-1 and nesfatin-1. Also, change in the serum irisin-1 and nesfatin-1 were correlated with the change in glycemic and anthropometric indices in addition to LDL-cholesterol. Also, exercise training significantly reduced fasting insulin and HOMA-IR in all the intervention groups.

**RCT Registration Code:** IRCT20180806040721N2.

## Introduction

It is generally accepted that exercise has important beneficial effects on metabolic disorders associated with impaired energy homeostasis (Algul et al., 2017). It is also known that a delicate balance exists in the ratio of energy expenditure to energy intake and that the molecular mechanism underlying this balance is complex (Hawley and Holloszy, 2009). Many critical processes, including those related to hormonal factors and the nervous system, play important roles in maintaining this balance (Algul et al., 2017). The importance of skeletal muscle and adipose tissue as secretory organs of many energy-regulating hormones is well-known (Luo and Liu, 2016). However, studies show that the level of these hormones changes after training sessions but the exact mechanisms by which exercise regulates energy homeostasis via energy-related hormones are still unclear.

The potential effects of exercise on altering the levels of hormones involved in energy regulation (particularly irisin-1 and nesfatin-1) is an interesting topic that has attracted the attention of many researchers (Oh et al., 2006; Norheim et al., 2014; Dianatinasab et al., 2020). Previous research identified nesfatin-1 as a new hypothalamic anorectic peptide, which is involved in metabolic regulation and food intake via a leptin-independent mechanism (Oh et al., 2006). However, studies on the effects of training on nesfatin-1 level provided contradicting findings, i.e., no change (Ghanbari-Niaki et al., 2010), decrease (Bajer et al., 2015), and increase (Ahmadizad et al., 2015), in the level of nesfatin-1. It was reported that the serum level of irisin-1 rises for a short period of time immediately after an exercise session (Fox et al., 2018). It has been reported that elevated irisin-1 levels in response to chronic exercise may reduce weight in obese individuals and insulin resistance in patients with diabetes (Bostrom et al., 2012; Kim et al., 2016; Whillier, 2020). In addition, serum irisin-1 has been found to have an inverse correlation with glucose intolerance (Choi et al., 2013). It is not clear whether training can increase the baseline serum irisin-1, as even the short-term increase in serum irisin-1 due to exercise may cause some beneficial metabolic changes (Wang et al., 2017, 2018). However, the mechanism of these changes seems to be complicated since it is revealed that the lipid tissue is responsible for the baseline level of irisin-1 in the blood among obese individuals (Huh et al., 2012). Furthermore, the serum irisin-1 level is positively correlated with higher waist circumference, body fat mass, and unfavorable lipid profile (Crujeiras et al., 2014; Polyzos et al., 2018). Irisin-1 is an exercise-related hormone that increases the rate of energy consumption and reflects the individual's metabolic status. Irisin-1 is an important agent for evaluating the effect of treatment of diseases associated with metabolic impairment including obesity and diabetes (Algul et al., 2017).

With regard to the above facts, it is important to clarify the effects of training on hormones such as nesfatin-1 and irisin-1 that are involved in the regulation of energy homeostasis (Bajer et al., 2015; Algul et al., 2017). A training program may be used as a non-pharmacological treatment to raise the levels of these hormones and improve the energy balance. It is suggested that exercise-induced increase in the levels of irisin-1 and nesfatin-1 may reduce food intake and increase energy expenditure (Dianatinasab et al., 2020).

Putting aside the conflicting results, regular physical activity is prescribed for patients with different metabolic diseases including diabetes, dyslipidemia, and metabolic syndrome (Amanat et al., 2020). Metabolic syndrome, as a cluster of health-related conditions including glucose intolerance, hypertension, dyslipidemia, and central obesity is an overwhelming health problem across the globe (Kaur, 2014). Regardless of the molecular pathways, regular exercise, especially a program with a combination of aerobic and resistance training can improve most conditions related to metabolic syndrome (AminiLari et al., 2017).

To the best of our knowledge, the effects of a various training program on serum nesfatin-1 and irisin-1 using a well-designed controlled clinical trial have not been studied yet. This study aimed to evaluate the effects of AE, RE, and CE on two different energy regulatory hormones, i.e., nesfatin-1 and irisin-1. Therefore, this study meant to examine two hypotheses: (i) whether different training regimens including aerobic, resistance, and a combination of aerobic and resistance, alter the serum level of irisin-1, nesfatin-1 and HOMA-IR, and (ii) whether changes in the serum irisin-1, nesfatin-1 levels, and HOMA-IR are associated with changes in other metabolic and anthropometric parameters in women with metabolic syndrome.

## Materials and Methods

### Participants

The study was carried out in accordance with the declaration of Helsinki, and it was approved by the local ethics committee of Shiraz University of Medical Sciences (approval code: IR-SUMS-REC: 97-01-04-17589). Details of the study participants and the study protocol are available elsewhere (Dianatinasab et al., 2020).

**Figure 1:**
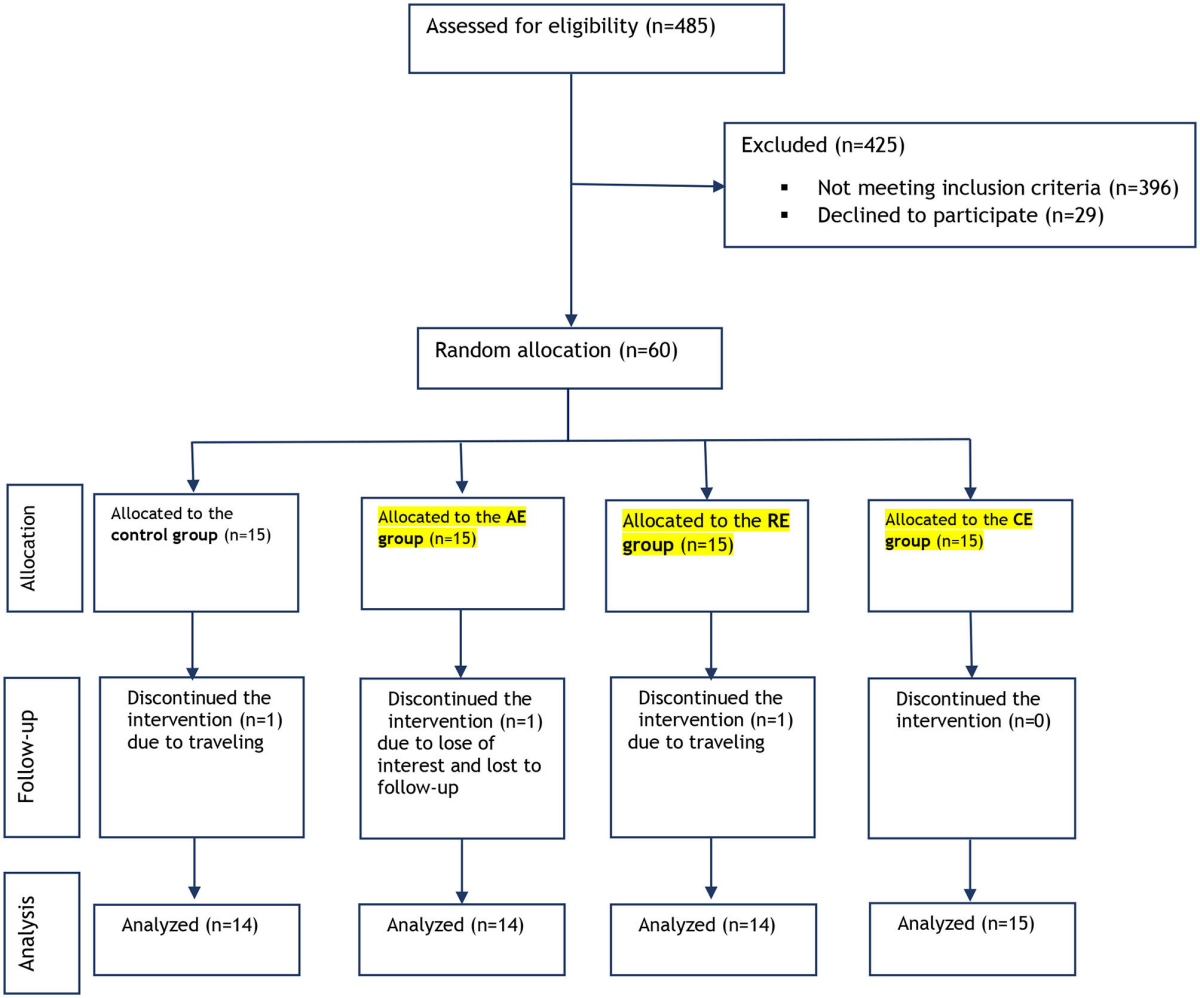
Study flow diagram.

We followed the CONSORT 2010 flow diagram (Figure 1) to conduct this randomized controlled trial in order to examine the effects of aerobic exercise (AE), resistant exercise (RE), and combined exercise (CE) on the levels of serum irisin-1, nesfatin-1, fasting plasma glucose (FPG), insulin, insulin resistance (HOMA-IR), lipid profile, and body composition in obese women with metabolic syndrome. Metabolic syndrome was confirmed when at least three of the following conditions were met: abdominal obesity (waist circumference >88 cm in women), triglyceride ≥150 mg/dL, HDL-cholesterol ≤ 50 mg/dL in women, hypertension (systolic blood pressure ≥130 mmHg and/or diastolic blood pressure ≥85 mmHg), and increased FPG (≥100 mg/dL) (Expert Panel on Detection, 2001). Inclusion criteria for subjects were as follows: having at least three diagnostic criteria of metabolic syndrome, aged between 46 and 60 years, being premonopause, sedentary lifestyle, non-smoker, no history of cancer, no history of cardiovascular and musculoskeletal disorders, no use of dietary or ergogenic supplements, and willingness to take part in the study. A sedentary lifestyle was defined as <600 MET min/week based on the short term of the International Physical Activity Questionnaire (IPAQ) (Moghaddam et al., 2012). Exclusion criteria were unwillingness to continue with the intervention (missing more than 20% of the training sessions), pregnancy, starting a specific dietary regimen and changing medication. All selected participants read and signed a written informed consent form (except for illiterate participants who gave a verbal informed consent).

### Study Design

The study protocol was registered with the Iranian Registry of Clinical Trials (IRCT) under the registration code of IRCT20180806040721N2. The study was conducted from February to June 2018 on 60 overweight/obese women with metabolic syndrome selected from women visiting a public clinic affiliated with Shiraz University of Medical Sciences, Iran. Block randomization with a block size of four was used for assigning the subjects into four groups: (I) AE, (II) RE, (III) CE, and (IV) the control group. Each group consisted of 15 women who were not blind to group assignments due to the nature of the intervention. However, neither the laboratory staff nor the data collectors or the biostatistician were aware of group assignments. The eligible women were contacted by a nurse to participate in the study. The women were informed of the study method and objectives and were asked to sign the informed consent form before the initiation of the study. The participants were examined by a general practitioner before commencing the study to ensure that the interventions would not jeopardize their health condition.

### Intervention: The Training Program

The control group was advised not to change their physical activity during the study. Also, their compliance with the interventions was monitored every other week via phone calls.

All the participants in the intervention groups engaged in group-based training sessions. In all the training groups each session was started with 10 min of stretching exercises and ended with 10 min of balance exercises.

The control group was advised not to change their physical activity during the study. Also, their compliance with the interventions was monitored every other week via phone calls.

Aerobic exercise (AE) consisted of running on the treadmill (Cosmuse/hp model Saturn,® Germany) three times per week on non-consecutive days. In the group-based training sessions, the exercise time was increased gradually from 30 to 60 min in each session after 2 weeks. Each 60-min session included the exact order of stretching exercises, treadmill walking (15–20 min), rest (5–10 min), stationary cycling (15–20 min), and balance exercises. The exercise intensity was based on the maximum heart rate (HR_max_) which was estimated for each participant with the following formula: HR_max_ = 208 − 0.7 × age (Tanaka et al., 2001). The intensity of training was started at 60% HR_max_ and was gradually elevated to 75% HR_max_ during each session. The heart rate was monitored using the wristband heart rate monitor. The accuracy of the wristband heart rate monitor was approved by comparing the results with those collected via electrocardiography.

Resistance training (RE): The exercise techniques were instructed to the women and their performance was supervised by professional trainers in group-based training sessions. The exercise was performed for 2 sessions per week (each session 60 min) during the first 2 weeks and was increased to 3 sessions on non-consecutive days per week. Forty minutes of strength training consisted of 2 sets of 10 different exercises, including bench press, seated row, shoulder press, chest press, lateral pull-down, abdominal crunches, leg press, leg extension, triceps pushdown, and seated bicep curls, for upper and lower parts of the body. All the resistance exercises were performed according to the National Strength and Conditioning Association guideline (Haff and Triplett, 2015).

The subjects had 8–10 repetitions for each exercise and 5–10 min of rest between each set. For the first 2 weeks of training, the intensity of the exercises was 60% one-repetition maximum (1RM) and was elevated to 75–80% 1RM from the 3rd week on. In order to estimate 1RM, participants were tested on the leg press and seated bicep curl for the upper and lower body at 2 non-consecutive days. After warm-up for leg press participants positioned in such that 90° knee flexion was created. A conservative weight was applied to the apparatus and participants were instructed to fully extend their knees and repeat the procedure until exhaustion. For seated bicep curl participants lifted the weight in the range of 100° to a full extension until exhaustion. Considering inexperience participants and the risk of injury, instead of direct measurement, the estimation of 1RM was performed for each participants using the Brzycki formula (Brzycki, 1993): 1RM = (Amount of displaced weight (kg)/((1.0278-(0.0278 × counts of repetitions))).

**Figure 2:**
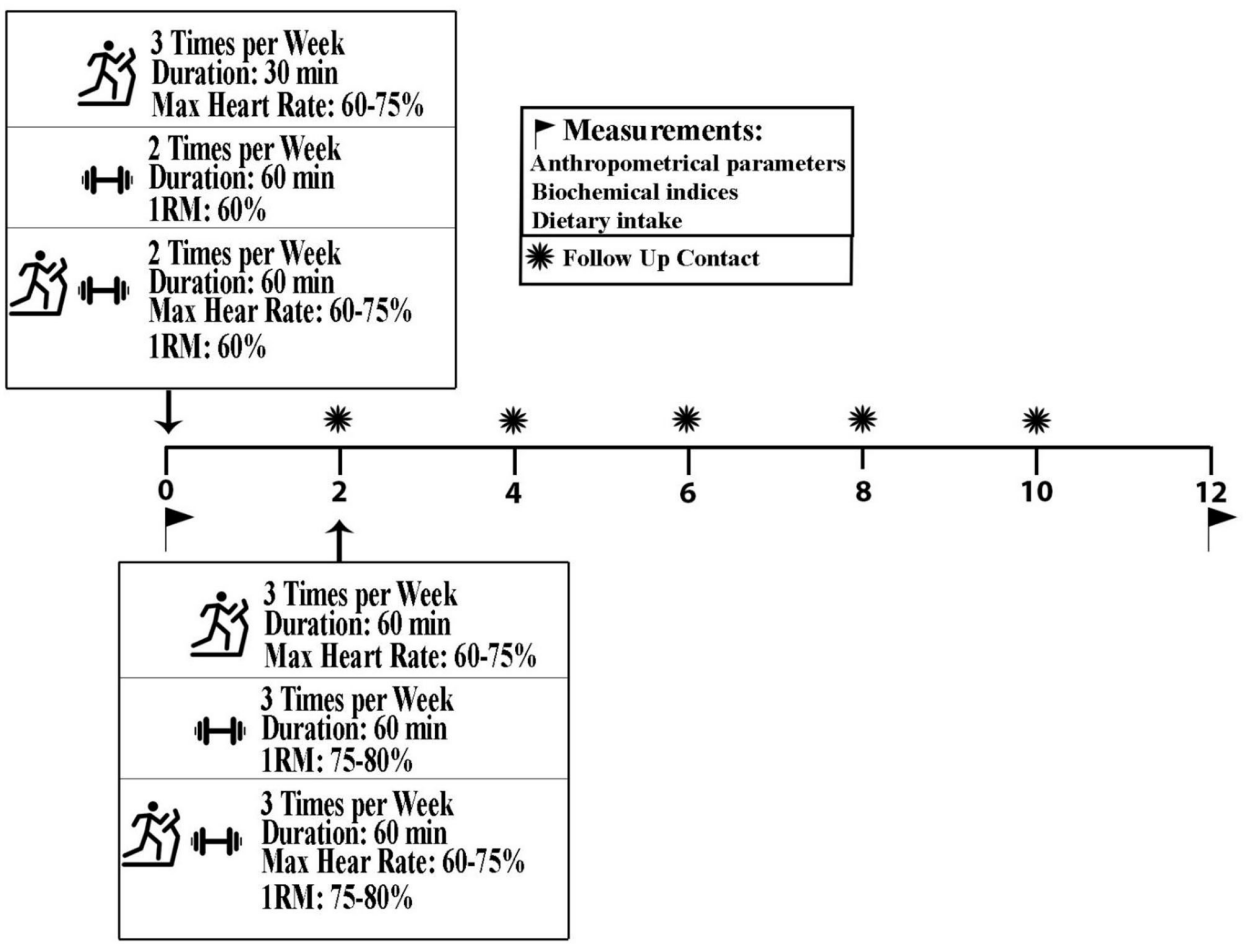
An overview of the study protocol.

Combined exercise (CE): This group also participated in group-based training sessions, so that they performed both AE and RE simultaneously in one session. The CE group performed exercise two sessions a week for the first 2 weeks and three sessions for the rest of the intervention period. Each session was consisting of 20 min of walking on a treadmill, followed by 5 min rest and one set of strength training, consisting of 10 different exercises similar to the RE exercise program. The intensities of the aerobic and strength exercises were gradually increased according to the AE and RE protocols, respectively. Figure 2 demonstrates an overview of the study protocol.

### Nutrition and Supplements

Before and after the intervention we evaluated dietary intake to collect data on dietary energy and macronutrient content using 24-h dietary recall. Data were entered into Nutritionist IV software (First Databank® Inc., Hearst Corp., San Bruno, CA) for analysis. Dietary intakes were presented as the amount of energy and macronutrients (protein, carbohydrate, fat, and fiber) per day for each participant. Eventually, these data were transferred to SPSS software for further analysis. The participants were also asked not to change their dietary habits during the study period. The participants were contacted every other week by a nutritionist to ensure that no major dietary changes occurred.

### Measurement

Before the study intervention, we collected demographic data and several anthropometric indexes including body weight, height, waist, and hip circumference. To calculate body mass index (BMI) and waist-to-hip ratio (WHR), we measured anthropometric measures in two mornings (before and after intervention) while the participants were wearing no shoes and minimal cloths according to the WHO guideline (World Health Organization, 1995). Body composition, including body fat percentage (%BF) and skeletal muscle mass (SMM) were measured using the bioelectric impedance analysis (InBody 720; BioSpace®, Co., Korea) with light clothing, in the standing position and no vigorous exercise session for at least 6 h before the measurement. To increase the precision of the assessment, participants were instructed to maintain normal hydration status. Blood pressure was measured in a seated position after 5 min rest and the average of the two consecutive measurements was recorded.

Before the intervention, 5 mL of venous blood was taken from each participant in the morning after 12 h of fasting. The samples were incubated for 15 min in the room temperature for clotting and then centrifuged for 10 min in 4,000 rpm to separate serum. The serum was frozen at −70°C until the time of laboratory analysis. The blood samples were also collected 24 h after the last exercise session and all the above-mentioned processes on blood samples were repeated. Each sample was tested once for biochemical analysis unless the result was unusually out of normal range to confirmation.

Fasting plasma glucose (FPG), total cholesterol, LDL-cholesterol, HDL-cholesterol, and triglyceride were measured with commercial kits (Pars Azmoon®, Tehran, Iran) and an autoanalyzer (Biotecnica instrument®, Rome, Italy) using the photometric and enzymatic methods. The serum fasting insulin and serum irisin-1 and nesfatin-1 levels were assessed by the enzyme-linked immunosorbent assay (ELISA) method and using the human insulin kit (ZellBio®, Ulm, Germany) and human irisin-1 and nesfatin-1 kit (Phoenix Pharmaceuticals®, Burlingame, USA). HOMA-IR was calculated using the following formula: HOMA-IR= [fasting glucose (mg/dL) × fasting insulin (mU/L)/405].

### Statistical Analysis

The sample size was calculated to detect the least-significant difference between the intervention groups in terms of the serum nesfatin-1 level after the intervention as large as 1.8 ng/ml (standard deviation: SD = 1.3). In addition, the alpha and power were set at 0.05 and 80%, respectively. Accordingly, the sample size was estimated as 15 participants per group (Huh et al., 2015; Shabani and Izaddoust, 2018). The Shapiro-wilk test was used to assess the normal distribution of data. The baseline measures of the individuals were compared using the one-way ANOVA test. Before and after intervention measures with normal and non-normal distributions were compared using Paired *t*-test and Mann-Whitney *U*-test, respectively. The effectiveness of exercise programs was examined by comparing the mean difference of changes in each variable using the ANCOVA test, adjusted for the baseline measures as a covariate, followed by the Bonferroni *post-hoc* test. We further checked the correlations between irisin-1 and nesfatin-1 with the study variables including. The significance level was set as *p* < 0.05, and the data analysis was performed using the IBM SPSS Statistics 25 (Armonk, NY: IBM Corp).

## Results

### Participants' Characteristics

Figure 1 presents the flow diagram of the current study. Among sixty women who met the inclusion criteria and accepted to participate, 57 completed the 12-week study period. The mean age (±SD) of participants was 54.5 (±6.9 years). Two (3.5%) of the participants were illiterate, 45 (79%) had a high school degree and 10 (17.5%) had a bachelor or higher degree. Participants had 4.1± 1.6 years of diabetes history (between 3 and 5 years) and they were using one or two daily doses of metformin (500 mg) plus a maximum one dose of glibenclamide (5 mg). None of the participants were smokers nor performed habitual exercise.

### Baseline Measurements and Dietary Intake

There was no significant difference in baseline measures between the study groups except for muscle mass that was less in CE group when compared with the other three intervention groups (*p* < 0.05). The results of the analysis of food intake are shown in Table 1. The comparison of reported food intakes before and after intervention did not show any significant change (*P* > 0.05). This was expected as participants were instructed not to change their dietary habits and food intake.

**Table 1:** Dietary intake at baseline and end of the interventions in the study groups.

**Marker**	**Control**			**AE**			**RE**			CE^*^		
	**Pre (Mean ± SD)**	**Post (Mean ± SD)**	**p-value^a^**	**Pre (Mean ± SD)**	**Post (Mean ± SD)**	**p-value^a^**	**Pre (Mean ± SD)**	**Post (Mean ± SD)**	**p-value^a^**	**Pre (Mean ± SD)**	**Post (Mean ± SD)**	***p*-value^a^**
Energy (kcal)	2,195 ± 138	2,217 ± 98	0.421	2,129 ± 199	2,321 ± 208	0.347	2,108 ± 172	2,142± 190	0.142	2,202 ± 182	2,281± 139	0.492
Protein (g)	81.70 ± 11.9	84.31 ± 9.4	0.461	80.91 ± 16.8	79.67 ± 14.2	0.513	74.12 ± 8.2	78.19 ± 8.6	0.232	86.86 ± 10.3	85.62 ± 12.4	0.500
Total fat (g)	66.35 ± 8.6	66.37 ± 9.0	0.512	65.42 ± 6.9	63.47 ± 9.8	0.280	68.83 ± 5.9	65.23 ± 5.1	0.114	69.43 ± 10.7	67.77 ± 7.9	0.263
Saturated fat (g)	14.2 ± 3.3	13.3 ± 5.3	0.413	18.86 ± 7.0	18.31 ± 8.1	0.149	16.22 ± 4.5	16.03 ± 4.9	0.502	16.1 ± 6.8	15.8 ± 5.5	0.788
Monounsaturated fat (g)	28.17 ± 6.4	28.43 ± 6.6	0.098	22.77 ± 7.6	24.19 ± 9.7	0.381	27.00 ± 12.2	26.98 ± 12.1	0.329	28.26 ± 12.7	27.06 ± 8.9	0.470
Polyunsaturated fat (g)	23.98 ± 6.8	24.64 ± 8.0	0.112	23.79 ± 8.9	20.97 ± 7.0	0.274	25.61 ± 9.0	22.22 ± 7.8	0.081	25.07 ± 7.4	24.91 ± 8.8	0.816
Carbohydrate (g)	339.91 ± 33.8	342.48 ± 43.4	0.308	339.19 ± 60.2	343.12 ± 41.20	0.202	321.42 ± 51.3	331.06 ± 46.0	0.068	300.85 ± 59.0	328.66 ± 53.6	0.147
Dietary fiber (g)	19.1 ± 6.3	19.4 ± 5.0	0.149	16.3 ± 2.9	17.10	0.371	15.8 ± 7.6	16.1 ± 6.3	0.215	18.0 ± 4.0	17.2 ± 3.3	0.322

a *p-value from the paired sample t-test comparing pre and post intervention dietary intake in each group*.

* *This group performed both AE and RE simultaneously in one session*.

### Anthropometric and Body Composition

The summary of pre and post measurements and the results of the comparison between groups are presented in Table 2. Accordingly, we observed no significant changes in the control group, whereas, both AE and CG experienced significant reductions in body weight and BMI (*p* < 0.05). In addition, all of the three training groups had significantly lower body fat percentage and higher muscle mass after 12 weeks of intervention (*p* < 0.05). Regarding WHR, none of the study groups exhibited significant changes. Analysis of variance of the post-intervention measures indicates that only CE group had a lower muscle mass at the end of intervention when compared to the control group (*p* < 0.05). However, based on the results of ANCOVA using pre-test values as a covariate, body weight, BMI, body fat percent, and muscle mass in the CE group were significantly different from the control group. In addition, compared to the control group, both AE and RE groups had significantly lower body fat and higher muscle mass, respectively. According to the results, the CE group achieved significantly better anthropometric indices and lipid profile when compared to the control group (Figure 3).

**Table 2:** Laboratory values, anthropometric measures in the groups at baseline and after 8 weeks of the interventions.

**Marker**	**Control (*****n*** **=** **14)**			**AE (*****n*** **=** **14)**			**RE (*****n*** **=** **14)**			**CE (*****n*** **=** **15)**			***P-*value^b^**	***P*-value^c^**	***P*-value^d^**
	**Pre (Mean ± SD)**	**Post (Mean ± SD)**	***P*-value^a^**	**Pre (Mean ± SD)**	**Post (Mean ± SD)**	***P*-value^a^**	**Pre (Mean ± SD)**	**Post (Mean ± SD)**	***P*-value^a^**	**Pre (Mean ± SD)**	**Post (Mean ± SD)**	***P*-value^a^**			
Weight (Kg)	72.50 ± 10.67	73.10 ± 10.78	0.098	74.21 ± 6.88	73.17 ± 8.10	0.020	73.71 ± 9.10	72.97 ± 8.79	0.012	72.86 ± 8.74	74.21 ± 9.03^*^	0.017	0.955	0.873	0.004
BMI (kg/cm^2^)	29.09 ± 4.61	29.59 ± 4.81	0.099	29.25 ± 4.03	28.554.69	0.019	28.97 ± 2.95	28.59 ± 2.98	0.003	30.25 ± 3.34	28.42 ± 3.2^*^	0.016	0.789	0.857	0.002
WHR	0.88 ± 0.05	0.89 ± 0.05	0.208	0.88 ± 0.05	0.84 ± 0.09	0.083	0.88 ± 0.05	0.82 ± 0.09	0.073	0.87 ± 0.03	0.84 ± 0.09	0.077	0.950	0.233	0.175
Body fat (%)	33.75 ± 2.61	34.47 ± 2.80	0.323	34.88 ± 3.23	33.66 ± 3.42^*^	0.007	34.05 ± 3.62	33.10 ± 4.02	0.007	33.84 ± 3.50	32.12 ± 3.34^*^	<0.001	0.787	0.310	0.003
Muscle mass (Kg)	39.54 ± 2.99	39.46 ± 2.46	0.704	39.82 ± 3.68	40.82 ± 3.30	0.017	39.61 ± 2.83	41.02 ± 2.89^*^	<0.001	35.73 ± 6.66	36.99 ± 6.44^*^	0.001	0.040	0.042	0.002
SBP (mmHg)	133.08 ± 11.12	135.12 + 11.09	0.893	136.40 ± 15.21	131.22 + 10.30	0.214	131.17 ± 9.68	131.78 + 13.59	0.781	131.68 + 11.47	127.65 + 10.05	0.141	0.234	0.553	0.294
DBP (mmHg)	87.31 ± 9.14	88.06 + 7.5	0.452	91.02 ± 10.11	88.50 + 6.48	0.191	93.55 ± 8.99	90.04 + 10.10	0.243	89.21 + 10.19	86.87 + 8.92	0.172	0.411	0.298	0.412
FPG (mg/dL)	163.35 ± 21.81	164.35 ± 23.57	0.459	157.92 ± 31.37	153.15 ± 31.59^*^	0.003	158.92 ± 31.03	155.71 ± 28.99	0.037	162.15 ± 27.09	155.58 ± 27.21^*^	<0.001	0.949	0.747	0.001
Insulin (μIU/mL)	10.34 ± 1.55	10.46 ± 1.70	0.514	10.62 ± 1.03	10.03 ± 0.91	0.004	10.66 ± 1.50	9.91 ± 1.56^*^	0.001	10.60 ± 1.35	9.05 ± 1.27^*^^Δ^^#^	<0.001	0.930	0.059	<0.001
HOMA-IR	4.18 ± 0.85	4.20 ± 0.99	0.779	4.11 ± 0.74	3.69 ± 0.77^*^	<0.001	4.13 ± 0.67	3.72 ± 0.68^*^	0.012	4.24 ± 0.95	3.48 ± 0.83^*^	<0.001	0.977	0.137	<0.001
TG (mg/dL)	175.63 ± 27.01	177.13 ± 29.05	0.311	193.21 ± 50.12	190.42 ± 50.37	0.017	209.28 ± 54.95	202.64 ± 49.81	0.066	199.92 ± 34.17	191.76 ± 35.04^*^	<0.001	0.216	0.464	0.016
LDL (mg/dL)	118.75 ± 32.25	120.59 ± 33.13	0.116	93.64 ± 16.86	91.57 ± 16.35	0.034	97.14 ± 34.37	94.71 ± 34.95	0.003	109.69 ± 35.94	104.38 ± 38.04^*^	0.003	0.130	0.083	0.001
HDL (mg/dL)	50.92 ± 11.71	52.50 ± 13.24	0.187	52.35 ± 10.42	56.92 ± 10.11	0.041	54.28 ± 12.28	56.64 ± 12.41	0.117	49.61 ± 8.93	53.69 ± 9.14	<0.001	0.693	0.665	0.415
Cholesterol (mg/dL)	184.85 ± 25.42	186.42 ± 27.02	0.303	189.85 ± 34.77	184.57 ± 34.52^*^	<0.001	197.28 ± 15.96	193.50 ± 16.44	0.030	166.92 ± 37.76	161.84 ± 37.56^*^	0.003	0.053	0.037	0.002
Irisin-1 (ng/mL)	9.14 ± 1.27	9.07 ± 1.41	0.687	8.81 ± 0.99	9.41 ± 1.08^*^	<0.001	9.42 ± 0.75	9.97 ± 1.12	<0.001	8.64 ± 0.87	9.47 ± 1.19^*^	<0.001	0.167	0.281	0.003
Nesfatin-1	8.71 ± 1.39	8.88 ± 1.37	0.146	8.82 ± 1.33	9.64 ± 1.13^*^	0.002	9.34 ± 1.63	10.42 ± 1.69^*^	0.007	9.57 ± 1.52	10.58 ± 1.33^*^	<0.001	0.350	0.007	0.001

a *Paired sample t-test*,

b *One-way ANOVA between pre-test measurements*,

c *One-way ANOVA between post-test measurements*,

d *Analysis of covariance of post-test measurements with pre-test measurements as covariates. BMI, Body Mass Index; WHR, Waist to Hip Ratio; SBP, systolic Blood Pressure; DBP, Diastolic Blood Pressure; FPG, Fasting Plasma Glucose; HOMIIR, Homeostatic Model Assessment for Insulin Resistance; HDL, High-Density Lipoprotein; LDL, Low-Density Lipoprotein; TG, Triglyceride*.

* *Significant difference vs. control group*.

Δ *Significant difference vs. aerobic group*.

# *Significant difference vs. resistance group*.

**Figure 3:**
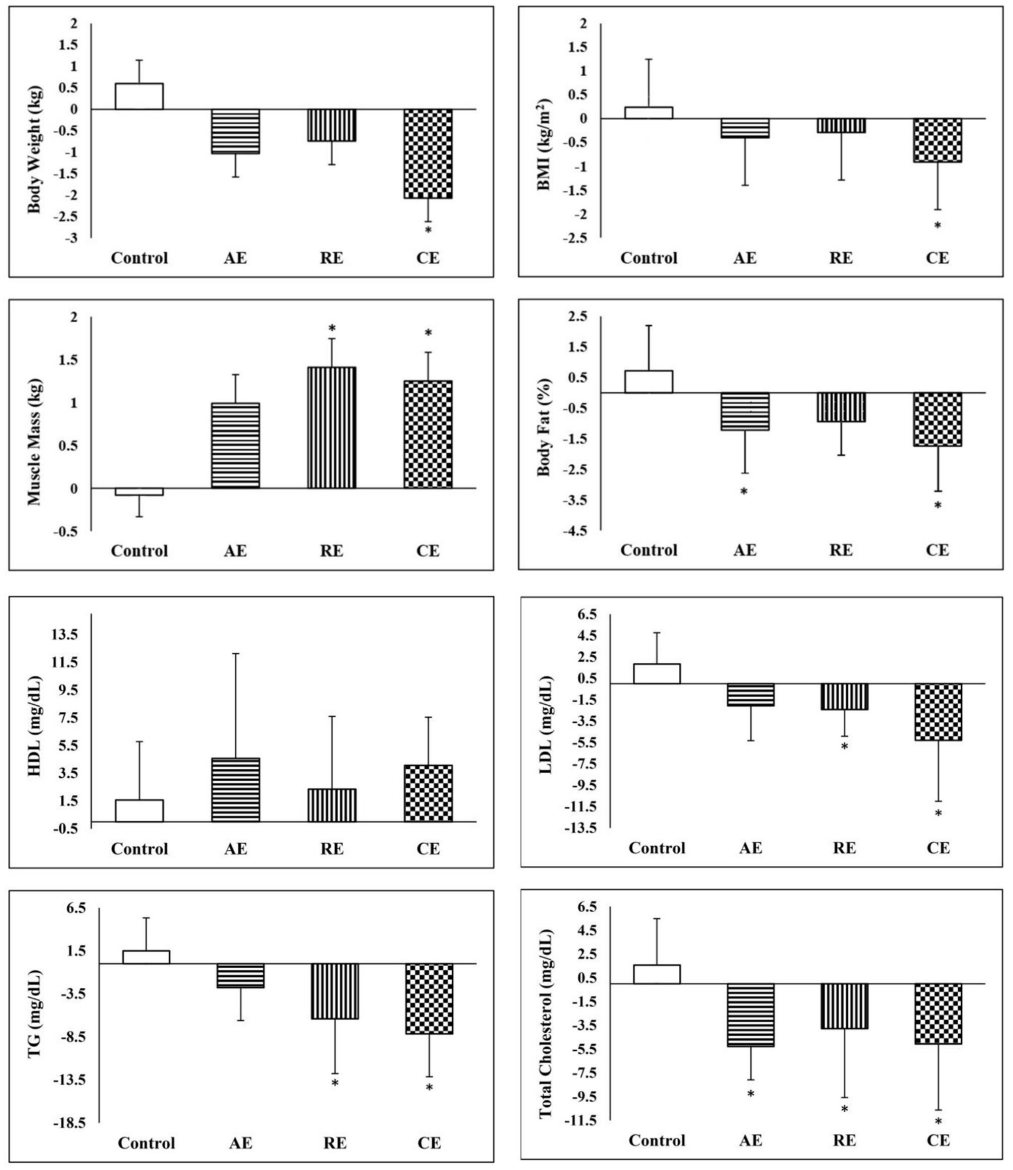
Comparison of changes in anthropometric and lipid profile between the groups. *Significant difference in comparison with the control group (*p* < 0.05). AE, aerobic exercise; RE, resistant exercise; CE, combined exercise.

### Glycemic and Lipid Indices

As expected, exercise training significantly reduced the FPG, fasting insulin and HOMA-IR in all intervention groups. However, the comparison of post-intervention measures between the study groups did not show any significant difference. Conversely, analysis of covariance demonstrated a significant reduction in FPG in CE and AE groups in comparison with the control group. When compared with the control group, the fasting serum insulin reduced significantly in the CE and RE groups. However, the reduction in CE (14%) group was even greater than what was observed in the AE and RE groups (5 and 7%, respectively). In addition, disregarding the exercise protocols, all the training groups exhibited significant reductions in insulin resistance in comparison with the control group. Both aerobic and combined exercises training programs improved lipid profile (TG, LDL, HDL, and total cholesterol) after 12 weeks of intervention. In the case of resistant exercise, the improvement was limited to LDL and total cholesterol. In comparison with the control group, the CE group experienced a higher reduction in TG, LDL, and total cholesterol. Nevertheless, a similar result was observed only in the reduction of total cholesterol in the AE group (Table 2).

### Irisin-1 and Nesfatin-1

**Figure 4:**
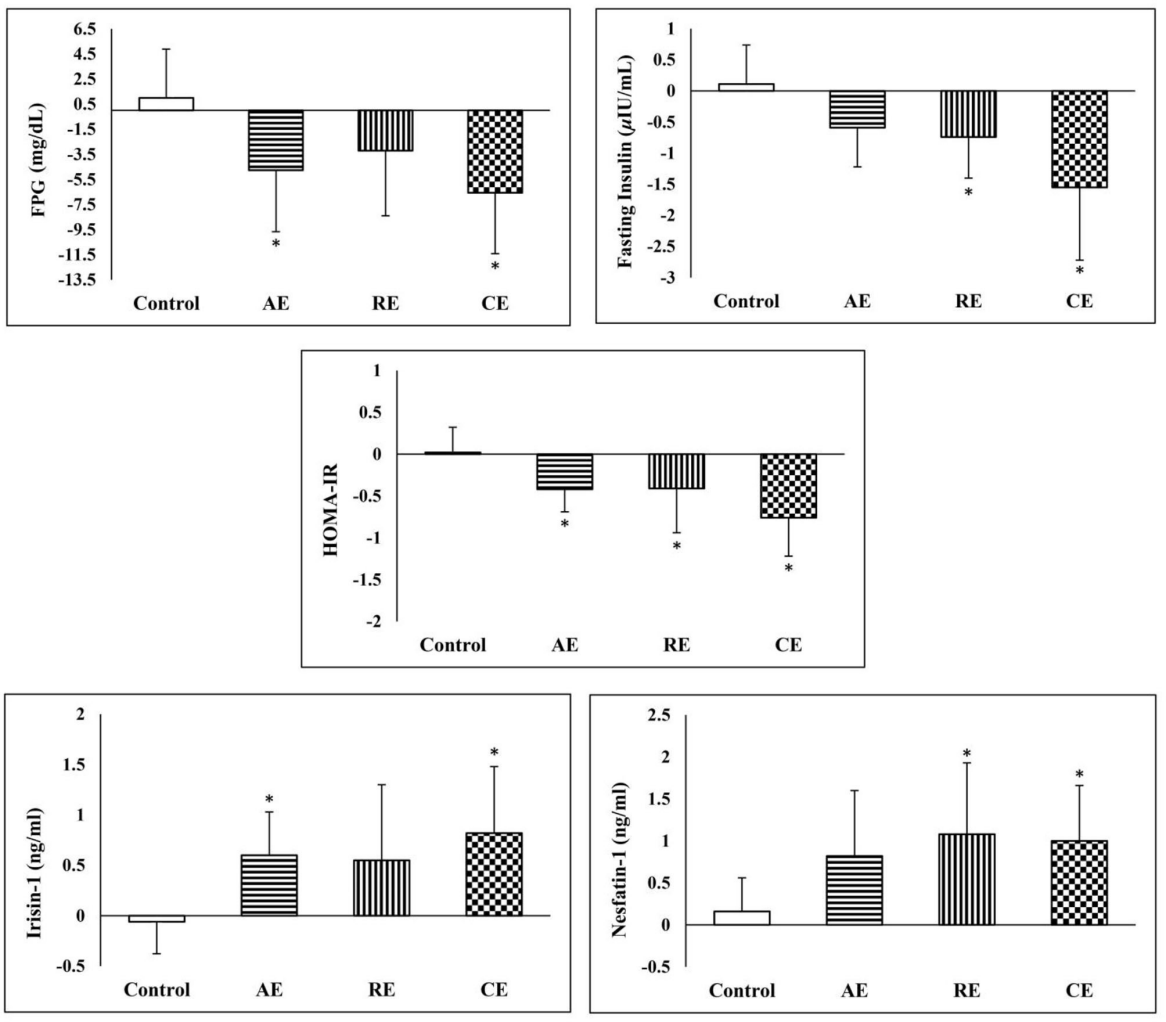
Comparison of changes in glycemic indices Irisin-1 and nesfatin-1 between the groups. *Significant difference in comparison with the control group (*p* < 0.05). AE, aerobic exercise; RE, resistant exercise; CE, combined exercise.

The serum concentration of irisin-1 and nesfatin-1 raised significantly after exercise training in three intervention groups except for irisin-1 in the RE group. The effectiveness of exercise on the increase of serum irisin-1 and nesfatin-1 remained significant after they were compared with the control group. As shown in Figure 4, the CE group had significantly better improvements in glycemic indices, serum irisin-1 and nesfatin-1 when compared with the control group.

Exploring the correlation between the observed changes in irisin-1, nesfatin-1 and anthropometric and metabolic indices among all the participants (Table 3) showed that irisin-1 was negatively and significantly correlated with body weight, BMI, body fat percent, fasting insulin, and HOMA-IR. Whereas, the increase in nesfatin-1 was significantly correlated with the reduction in body fat percent and LDL-cholesterol.

**Table 3:** Correlation between irisin-1/nesfatin-1 and metabolic and anthropometric parameters.

**Markers**	**Weight**	**BMI**	**WHR**	**Body fat**	**Muscle**	**FPG**	**Insulin**	**HOMI-IR**	**TG**	**LDL**	**HDL**	**Cholesterol**	**Irisin-1**	**Nesfatin-1**
Irisin−1	−0.271^*^	−0.278^*^	−0.141	−0.377^**^	0.200	−0.256	−0.264^*^	−0.337^*^	−0.067	−0.157	−0.094	−0.245	1	0.242
Nesfatin−1	−0.169	−0.185	−0.118	−0.0265^*^	0.020	−0.155	−0.178	−0.120	−0.037	−0.328^*^	−0.046	−0.069	0.242	1

*BMI, Body Mass Index; WHR, Waist to Hip Ratio; FPG, Fasting Plasma Glucose; HOMIIR, Homeostatic Model Assessment for Insulin Resistance; HDL, High-Density Lipoprotein; LDL, Low-Density Lipoprotein; TG, Triglyceride*.

* *Significant correlation at the 0.05 level*.

** *Significant correlation at the 0.01 level*.

## Discussion

This study aimed to compare the effects of 12 weeks of aerobic, resistance, and the combination of aerobic and resistance exercises on the serum levels of nesfatin-1 and irisin-1, anthropometric and glycemic indices, and lipid profiles of women with metabolic syndrome. The main finding of this randomized controlled trial was that the exercises in all intervention groups improved the serum concentration of irisin-1 and nesfatin-1. Except for serum irisin-1 in the RE group the effect of the exercise programs on the improvement in serum irisin-1 and nesfatin-1 was significant (when compared with the control group). We also found that serum irisin-1 was negatively and significantly correlated with body weight, BMI, body fat percent, fasting insulin, and HOMA-IR. Whereas, the increase of nesfatin-1 was correlated with a reduction of body fat percent and LDL-cholesterol. Accordingly, exercise training in all intervention groups reduced post-test FPG, fasting insulin and HOMA-IR. Also, a significant reduction in body weight and BMI was observed in the AE and CG groups.

The underlying mechanism of the exercise-induced nesfatin-1 response is not well-documented. Nesfatin-1 is synthesized in the central and peripheral nervous system as well as in the adipose tissue and is subsequently released into the blood circulation (Mogharnasi et al., 2019b). Nesfatin-1 exerts various actions at the level of nervous and digestive systems and plays a role in stress response and thermogenesis. It is believed that nesfatin-1 not only overwhelms appetite and therefore food intake but also it may affect energy expenditure and glucose homeostasis (Huang et al., 2008). By controlling the dietary habits of individuals, we found that 12-weeks exercise training increases nesfatin-1 among all three intervention groups. It has been hypothesized that nesfatin-1 modifies glucose homeostasis by mechanisms that increase glucose uptake and insulin sensitivity (Li et al., 2013). A previous study demonstrated that serum nesfatin-1 and insulin sensitivity in obese men were increased after upper-body resistance exercise was conducted (Mogharnasi et al., 2019b). Altogether, our results are consistent with the previous studies that showed an upsurge in nesfatin-1 and insulin sensitivity following high-intensity training (Ahmadizad et al., 2015; Ozcelik et al., 2018; Mogharnasi et al., 2019b). Moreover, another study reported that the reduction in nesfatin-1 induced by a high-fat diet is suppressed by the running wheel in mice (Chaolu et al., 2011). Nesfatin-1 reduces blood glucose by activating AMP-activated protein kinase, up-regulating the phosphorylation of protein kinase B in the pancreas, and by increasing the glucose transporter-4 membrane translocation in skeletal muscle and adipose tissue that in turn improves insulin sensitivity (Le et al., 2016). In line with our findings, a study suggested that nesfatin-1, weight, and fat percentage are likely to be improved significantly in response to a long-term exercise (Yazici, 2015). On the other hand, increased levels of the adipocytokine induced change by exercise can act as stimuli in reducing visceral and subcutaneous fat. In accordance with our results, a negative correlation has been reported between nesfatin-1 and variables such as body fat percentage by a few previous studies (Tsuchiya et al., 2010; Mogharnasi et al., 2019b). In addition, it has been reported that 8 weeks of moderate-intensity aerobic exercise diminished BMI and insulin and raised serum levels of nesfatin-1 in obese women (Nabil and El Sayyad, 2015). As noted in the literature, nesfatin-1 is highly sensitive to long-term training and significant loss of weight and fat percentage (Nabil and El Sayyad, 2015; Mogharnasi et al., 2019a).

Secretion of nesfatin-1 may contribute to the treatment of obesity and diabetes because of its anorexigenic and antihyperglycemic effects and even it may have effects on depression and anxiety (Schalla and Stengel, 2018). These pieces of evidence suggest that long term exercise training might be an effective way to increase nesfatin-1. Because of these beneficial effects, nesfatin-1 may be a potential regulatory agent that exerts therapeutic effects of exercise training on some chronic diseases including obesity, type 2 diabetes, and metabolic syndrome (Schalla and Stengel, 2018; Öztürk Özkan G., 2020).

Previous studies have examined the effects of different exercise programs on serum irisin-1. Nevertheless, their results are not convincedly consistent to warrant a firm conclusion. We previously found that an 8-week exercise training program resulted in a non-significant change in circulating irisin-1 (Dianatinasab et al., 2020). Interestingly, after 12 weeks of intervention, we found that long term exercise program may have a direct effect on serum levels of irisin-1. In line with the findings of the current study, Huh et al. (2015) suggested that regardless of the participants' age, fitness level, or health status, different exercise regimens, including high-intensity swimming, treadmill, and resistance exercise, increase the level of circulating irisin-1. Similarly, Daskalopoulou et al. (2014) reported that three different exercise protocols significantly increased serum irisin-1 serum level, especially after the maximal exercise workload.

Comparing the results of previous studies with our findings, Kim et al. (2016) found that circulating irisin-1 level was increased by 8 weeks of resistance training in overweight/obese adults. They suggested that resistance training is beneficial to overweight/obese individuals. In line with the findings of the present study, the authors reported a significant change in body composition concomitant with an increase in serum irisin-1 level.

There are also reported that neither of aerobic, resistance, or combined exercises did increase serum irisin-1 levels (Dianatinasab et al., 2020). It should be noted that the reported non-significant change in serum irisin-1 may be due to the difference in the training regimens (60 min, 5 times/week vs. 60 min, 3 times/week).

Norheim et al. reported that the serum level of irisin-1 was increased right after 45 min of ergometer cycling and dropped 2 h after the exercise. Since the production of irisin-1 depends on PGC-1α activity and FNDC5 transcription, during an acute exercise, the sharp increase in serum irisin-1 could be due to FNDC5 mRNA translation rather than FNDC5 transcription (Norheim et al., 2014). As Norheim et al. declared, PGC-1α transcription and FNDC5 mRNA expression increased significantly after 12 weeks of systematic exercise, but these changes did not translate to a higher concentration of serum irisin-1. These discrepant results could be due to differences in study designs as Norheim et al. prescribed training exercise for both control and intervention groups and the blood samples were collected immediately and 2 h after training sessions.

In addition to acute or chronic exercise interventions, patients' anthropometric and health status may also affect the results. In our study, the female participants were overweight and obese and were diagnosed with metabolic syndrome. Similarly, the participants in a study by Norheim et al. were obese and prediabetic volunteers, but Pekkala et al. (2013) focused on healthy men, and their findings did not confirm the positive effects of AE, RE, and CE on irisin-1. Timmons et al. studied the effects of endurance and resistance exercise for 6 weeks on two different groups, including healthy individuals and those with type II diabetes. The results showed greater expression of FNDC5 in the intervention group compared with that of the control group (Timmons et al., 2012). Although Osella et al. (2018) examined the effects of different diets on the serum irisin-1 concentration and found that vegetable proteins and saturated fatty acids were positively associated with serum irisin-1. The authors did not examine the effects of exercise on serum irisin-1 concentration. To address the above issue, our study used a dietary regimen based on the pre-intervention dietary intake to adjust the effect of dietary components on serum irisin-1.

As expected, the exercise interventions induced a positive change in glycemic indices, and the change was bigger in the CE group. Our results were similar to those of a previous study that evaluated the efficacy of different exercise regimens on serum omentin-1 in diabetic women (AminiLari et al., 2017). The Pearson correlation showed that changes in the HOMA-IR, body weight and body fat percentage were correlated negatively with serum irisin-1 in all participants. Positive relationships between serum irisin-1 and metabolic disorders, such as obesity, diabetes, and cardiovascular diseases, are reported by previous studies (Sesti et al., 2014; Shoukry et al., 2016). Stengel et al. (2013) showed that obese patients had a higher level of circulating irisin-1 than normal subjects. In addition, serum irisin-1 was negatively correlated with fat mass and insulin resistance. Our study showed that the reduction in insulin resistance or body fat percentage is associated with higher serum irisin-1. Crujeiras et al. (2014) reported that the circulating irisin-1 was increased proportionally to the weight regain after patients underwent a calorie-restricted weight reduction diet.

There are two hypotheses to explain the underlying mechanisms behind the association of serum irisin-1 and weight gain. In the first hypothesis, an increase in the serum irisin-1 is an adaptive response to weight gain and an increase in body fat. Accordingly, the conversion of white fat tissue to brown adipose tissue can increase thermogenesis and energy consumption, and eventually, lead to weight loss through myokine irisin-1 (Bostrom et al., 2012). Irisin-1 stimulates the expression and activity of uncoupling protein1 (UCP1) and causes the browning of the white adipose tissue. Therefore, it increases thermogenesis and total energy expenditure that in turn, can reduce obesity (O'Neill and O'Driscoll, 2015; Zhang et al., 2016). This hypothesis was supported by the results of an animal study, in which irisin-1 improved hyperinsulinemia and glucose tolerance in mice with a high-fat diet (Zhang et al., 2014). In the second hypothesis, an increase in serum irisin-1 in adiposity may induce irisin-1 resistance. Irisin-1 is supposed to improve UCP1 production and promote the browning of white adipose tissues. However, it is contradictory that normal weight or anorexic patients have lower levels of circulating irisin-1 than obese individuals. While adipose tissue itself secretes irisin-1, an increase in the body fat and irisin-1 secretion does not lead to expected beneficial effects and leptin resistance (Sahin-Efe et al., 2018).

In the present study, a negative and significant correlation between change in irisin-1 concentration and HOMA-IR was observed. Similar to body fat and BMI, cross-sectional studies have reported a positive association between serum irisin-1 and insulin resistance (Hee Park et al., 2013; Sesti et al., 2014). At first sight, these results contravene our results, however, when considering the correlation between change in irisin-1 and glycemic status, there are several resembling studies. Yang et al. have shown that administration of irisin-1 to murine myocytes under an insulin-resistant state recovers insulin action (Yang et al., 2015). Reinehr et al. also have reported that in children who underwent lifestyle changes, the change in serum irisin-1 was reversely associated with fasting glucose and 2-h glucose of oral glucose tolerance test (Reinehr et al., 2015). Finally, it should be mentioned that there are debates on the accuracy of commercial ELISA kits to detect irisin-1. The developed antibodies for irisin-1 in these kits may bind to other proteins with a similar pattern too that could compromise the precision of the measurements. On the other hand, alternate methods such as mass spectrometry are more specificity but add more steps to sample preparation which adds uncontrolled deviation to the measurements (Albrecht et al., 2020).

Besides the evidence of improvement in the lipid profile in the training groups, no significant correlation was observed between these changes and serum irisin-1 and nesfatin-1, with one exception for a negative correlation between nesfatin-1 and LDL-cholesterol. While previous studies have shown a positive correlation between lipid profile and irisin-1 concentration in adolescents and adults (Shoukry et al., 2016; Jang et al., 2017). Contrary to recent studies that used a cross-sectional design, in the present study we conducted a randomized control trial to examine the correlations between the changes in irisin-1/nesfatin-1 concentration and serum lipids. To the best of our knowledge, no other RCTs have investigated the relationship.

### Study Strengths and Limitations

As a limitation, the inclusion of the expression of the FND5 gene in skeletal muscle and fat mass could reveal the source of circulating irisin-1 in the body. Also, we recruited women from a specific age range that may influence the generalizability of our results to the whole population. Accordingly, our results should be interpreted with caution when other age groups are considered. On the other hand, the process of evaluating the dietary intake before and after the intervention and using a dietary regimen based on the pre-intervention intake improved the quality of data collection and can be considered a strength of this study. So, a strength of the study is that it was diet-controlled. Besides, we asked (and monitored) the participants not to change their dietary habits during the study.

## Conclusions

We found that 12 weeks of training resulted in an increase in irisin-1 in the AE and CE groups and an increase in nesfatin-1 in all the intervention groups. Moreover, all the trained groups exhibited a positive alteration in anthropometric and glycemic indices and lipid profile in comparison with the control group. As nesfatin-1 and irisin-1 play many vital roles in glucose homeostasis as a regulator of glucose levels and energy expenditure, taking advantage of our results, and considering these beneficial effects, exercise training may be a safe and accessible strategy for most patients with metabolic syndrome to improve the effect of irisin-1 and nesfatin-1 on metabolic factors. Besides, the inconsistent findings on the effect of exercise in serum irisin-1 levels highlight the need for larger-scale RCTs on healthy and non-healthy participants.

## Data Availability Statement

The raw data supporting the conclusions of this article will be made available by the authors, without undue reservation.

## Ethics Statement

The studies involving human participants were reviewed and approved by Ethics Committee of Shiraz University of Medical Sciences. The patients/participants provided their written informed consent to participate in this study.

## Author Contributions

SA, ES, and AD co-designed the study and surveyed the materials. MP and RM were involved in data collection. SA, MF, AD, and ZB-H were responsible for conducting the statistical analyses, interpreting the data, and drafting the manuscript. SA and MF revised the manuscript critically for important content. All authors approved the final manuscript to be submitted and published.

## Conflict of Interest

The authors declare that the research was conducted in the absence of any commercial or financial relationships that could be construed as a potential conflict of interest.

## Abbreviations

MetS: Metabolic Syndrome
AE: Aerobic Exercise
RE: Resistant Exercise
CE: Combined Exercise
HOMA-IR: Homeostatic Model Assessment for Insulin Resistance
FPG: Fasting plasma glucose
SD: Standard Deviation
BMI: Body Mass Index
WHR: Waist-to-Hip Ratio
HDL: High-Density Lipoprotein
LDL: Low-Density Lipoprotein
TG: Triglyceride
UCP1: Uncoupling Protein-1
FNDC5: Fibronectin Type III Domain-Containing Protein 5.
